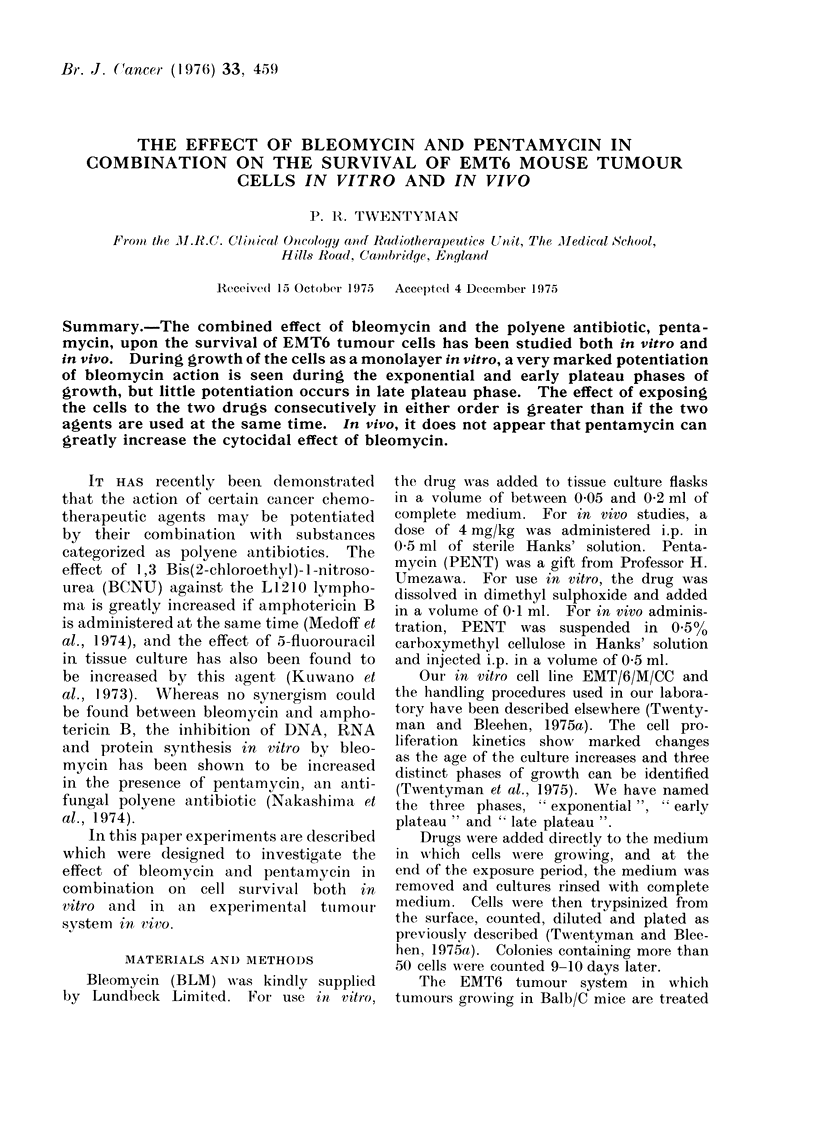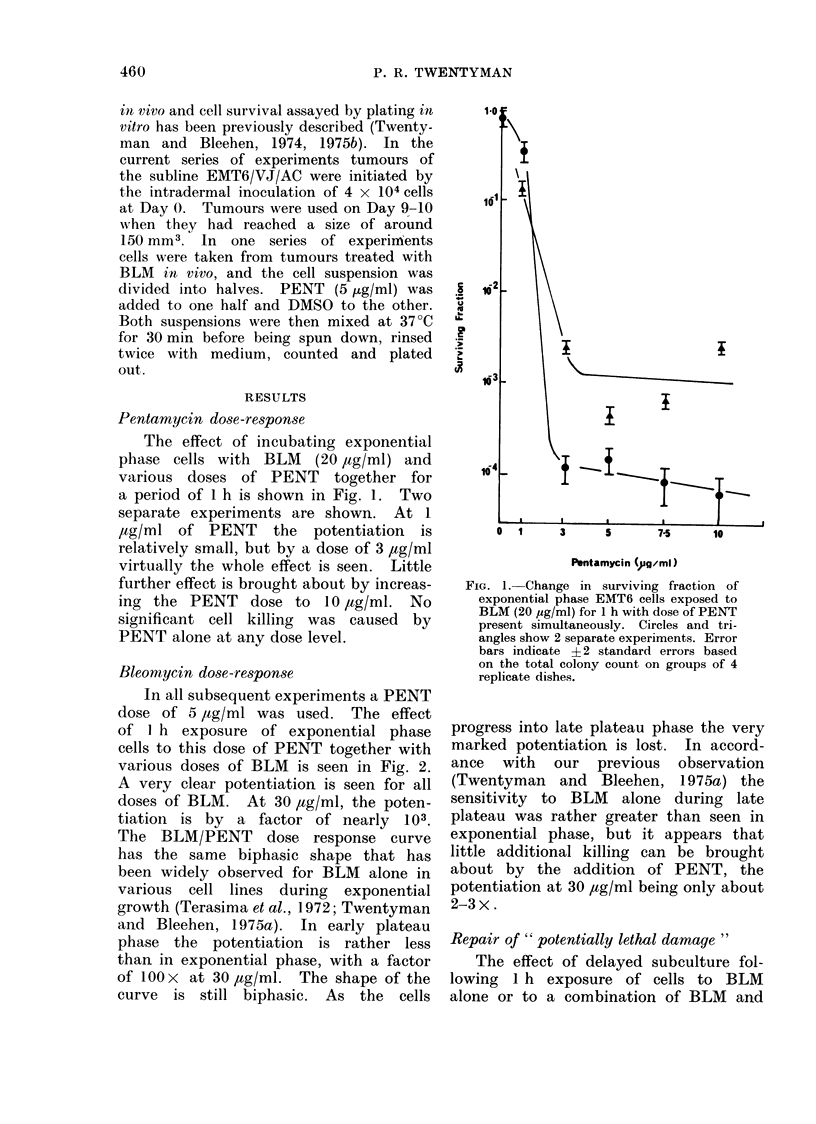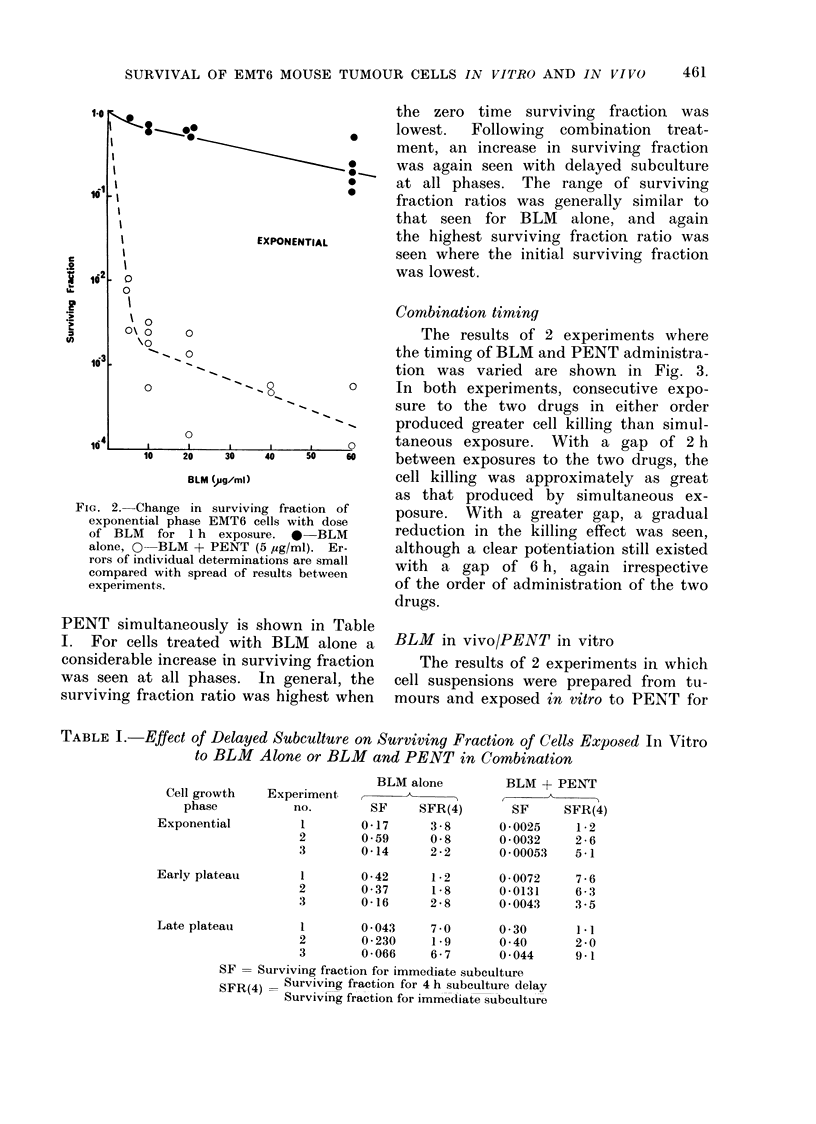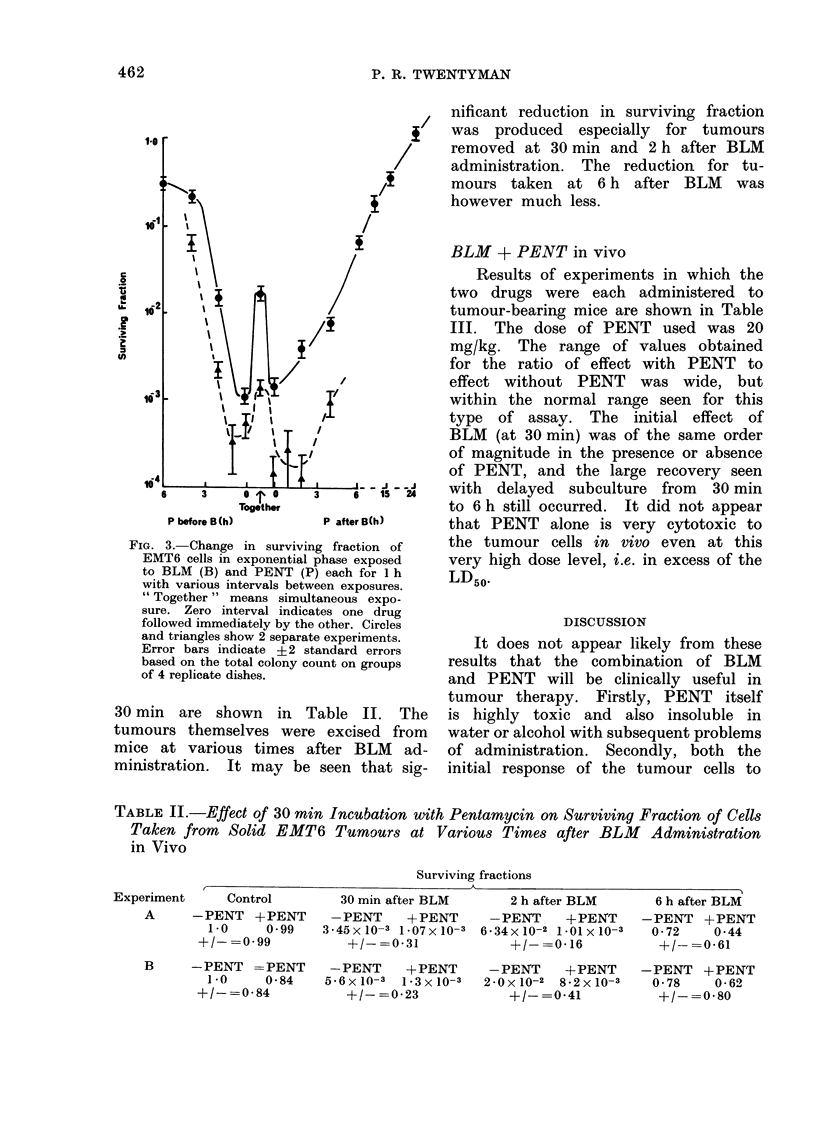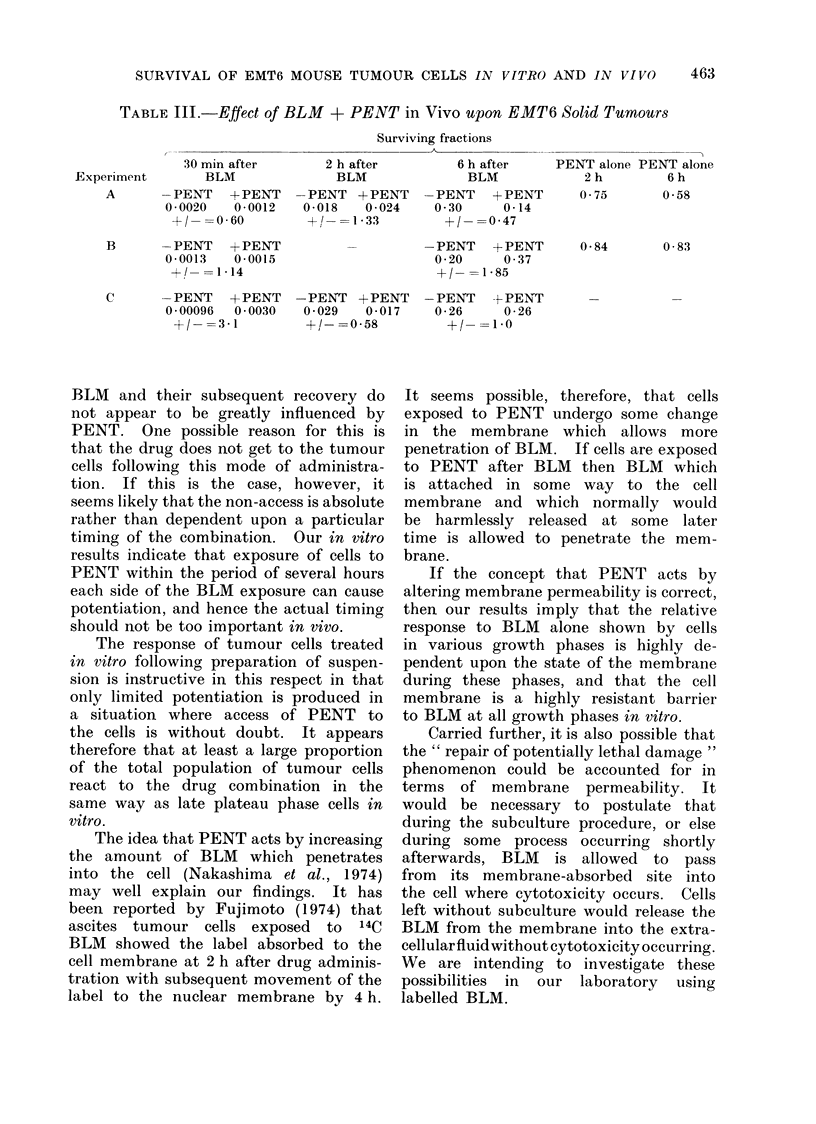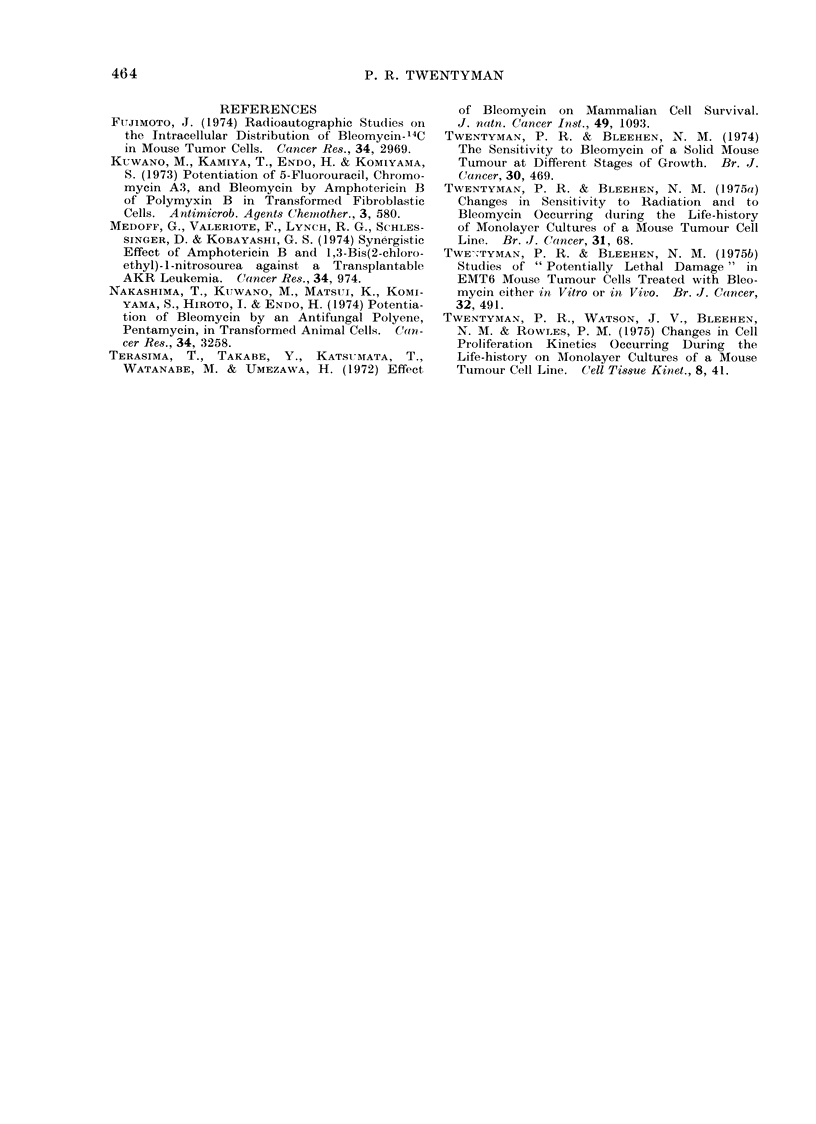# The effect of bleomycin and pentamycin in combination on the survival of EMT6 mouse tumour cells in vitro and in vivo.

**DOI:** 10.1038/bjc.1976.71

**Published:** 1976-04

**Authors:** P. R. Twentyman

## Abstract

The combined effect of bleomycin and the polyene antibiotic, pentamycin, upon the survival of EMT6 tumour cells has been studied both in vitro and in vivo. During growth of the cells as a monolayer in vitro, a very marked potentiation of bleomycin action is seen during the exponential and early plateau phases of growth, but little potentiation occurs in late plateau phase. The effect of exposing the cells to the two drugs consecutively in either order is greater than if the two agents are used at the same time. In vivo, it does not appear that pentamycin can greatly increase the cytocidal effect of bleomycin.


Br. J. Cancer (1976) 33, 459

### THE EFFECT OF BLEOMYCIN AND PENTAMYCIN IN COMBINATION ON THE SURVIVAL OF EMT6 MOUSE TUMOUR CELLS *IN VITRO* AND *IN VIVO*

P. R. TWENTYMAN

*From the M.R.C. Clinical Oncology and Radiotherapeutics Unit, The Medical School, Hills Road, Cambridge, England*

Received 15 October 1975 Accepted 4 December 1975

**Summary.—The combined effect of bleomycin and the polyene antibiotic, pentamycin, upon the survival of EMT6 tumour cells has been studied both *in vitro* and *in vivo*. During growth of the cells as a monolayer *in vitro*, a very marked potentiation of bleomycin action is seen during the exponential and early plateau phases of growth, but little potentiation occurs in late plateau phase. The effect of exposing the cells to the two drugs consecutively in either order is greater than if the two agents are used at the same time. *In vivo*, it does not appear that pentamycin can greatly increase the cytocidal effect of bleomycin.**

It has recently been demonstrated that the action of certain cancer chemotherapeutic agents may be potentiated by their combination with substances categorized as polyene antibiotics. The effect of 1,3 Bis(2-chloroethyl)-1-nitrosourea (BCNU) against the L1210 lymphoma is greatly increased if amphotericin B is administered at the same time (Medoff *et al.*, 1974), and the effect of 5-fluorouracil in tissue culture has also been found to be increased by this agent (Kuwano *et al.*, 1973). Whereas no synergism could be found between bleomycin and amphotericin B, the inhibition of DNA, RNA and protein synthesis *in vitro* by bleomycin has been shown to be increased in the presence of pentamycin, an antifungal polyene antibiotic (Nakashima *et al.*, 1974).

In this paper experiments are described which were designed to investigate the effect of bleomycin and pentamycin in combination on cell survival both *in vitro* and in an experimental tumour system *in vivo*.

### MATERIALS AND METHODS

Bleomycin (BLM) was kindly supplied by Lundbeck Limited. For use *in vitro*, the drug was added to tissue culture flasks in a volume of between 0·05 and 0·2 ml of complete medium. For *in vivo* studies, a dose of 4 mg/kg was administered i.p. in 0·5 ml of sterile Hanks' solution. Pentamycin (PENT) was a gift from Professor H. Umezawa. For use *in vitro*, the drug was dissolved in dimethyl sulphoxide and added in a volume of 0·1 ml. For *in vivo* administration, PENT was suspended in 0·5% carboxymethyl cellulose in Hanks' solution and injected i.p. in a volume of 0·5 ml.

Our *in vitro* cell line EMT/6/M/CC and the handling procedures used in our laboratory have been described elsewhere (Twentyman and Bleehen, 1975*a*). The cell proliferation kinetics show marked changes as the age of the culture increases and three distinct phases of growth can be identified (Twentyman *et al.*, 1975). We have named the three phases, " exponential ", " early plateau " and " late plateau ".

Drugs were added directly to the medium in which cells were growing, and at the end of the exposure period, the medium was removed and cultures rinsed with complete medium. Cells were then trypsinized from the surface, counted, diluted and plated as previously described (Twentyman and Bleehen, 1975*a*). Colonies containing more than 50 cells were counted 9–10 days later.

The EMT6 tumour system in which tumours growing in Balb/C mice are treated

*in vivo* and cell survival assayed by plating *in vitro* has been previously described (Twentyman and Bleehen, 1974, 1975*b*). In the current series of experiments tumours of the subline EMT6/VJ/AC were initiated by the intradermal inoculation of $4 \times 10^4$ cells at Day 0. Tumours were used on Day 9–10 when they had reached a size of around 150 mm$^3$. In one series of experiments cells were taken from tumours treated with BLM *in vivo*, and the cell suspension was divided into halves. PENT (5 μg/ml) was added to one half and DMSO to the other. Both suspensions were then mixed at 37 °C for 30 min before being spun down, rinsed twice with medium, counted and plated out.

### RESULTS

### *Pentamycin dose-response*

The effect of incubating exponential phase cells with BLM (20 μg/ml) and various doses of PENT together for a period of 1 h is shown in Fig. 1. Two separate experiments are shown. At 1 μg/ml of PENT the potentiation is relatively small, but by a dose of 3 μg/ml virtually the whole effect is seen. Little further effect is brought about by increasing the PENT dose to 10 μg/ml. No significant cell killing was caused by PENT alone at any dose level.

### *Bleomycin dose-response*

In all subsequent experiments a PENT dose of 5 μg/ml was used. The effect of 1 h exposure of exponential phase cells to this dose of PENT together with various doses of BLM is seen in Fig. 2. A very clear potentiation is seen for all doses of BLM. At 30 μg/ml, the potentiation is by a factor of nearly $10^3$. The BLM/PENT dose response curve has the same biphasic shape that has been widely observed for BLM alone in various cell lines during exponential growth (Terasima *et al.*, 1972; Twentyman and Bleehen, 1975*a*). In early plateau phase the potentiation is rather less than in exponential phase, with a factor of 100× at 30 μg/ml. The shape of the curve is still biphasic. As the cells

Fig. 1.—Change in surviving fraction of exponential phase EMT6 cells exposed to BLM (20 μg/ml) for 1 h with dose of PENT present simultaneously. Circles and triangles show 2 separate experiments. Error bars indicate ±2 standard errors based on the total colony count on groups of 4 replicate dishes.

progress into late plateau phase the very marked potentiation is lost. In accordance with our previous observation (Twentyman and Bleehen, 1975*a*) the sensitivity to BLM alone during late plateau was rather greater than seen in exponential phase, but it appears that little additional killing can be brought about by the addition of PENT, the potentiation at 30 μg/ml being only about 2–3×.

### *Repair of "potentially lethal damage"*

The effect of delayed subculture following 1 h exposure of cells to BLM alone or to a combination of BLM and

FIG. 2.—Change in surviving fraction of exponential phase EMT6 cells with dose of BLM for 1 h exposure. ●—BLM alone, ○—BLM + PENT (5 µg/ml). Errors of individual determinations are small compared with spread of results between experiments.

PENT simultaneously is shown in Table I. For cells treated with BLM alone a considerable increase in surviving fraction was seen at all phases. In general, the surviving fraction ratio was highest when the zero time surviving fraction was lowest. Following combination treatment, an increase in surviving fraction was again seen with delayed subculture at all phases. The range of surviving fraction ratios was generally similar to that seen for BLM alone, and again the highest surviving fraction ratio was seen where the initial surviving fraction was lowest.

### *Combination timing*

The results of 2 experiments where the timing of BLM and PENT administration was varied are shown in Fig. 3. In both experiments, consecutive exposure to the two drugs in either order produced greater cell killing than simultaneous exposure. With a gap of 2 h between exposures to the two drugs, the cell killing was approximately as great as that produced by simultaneous exposure. With a greater gap, a gradual reduction in the killing effect was seen, although a clear potentiation still existed with a gap of 6 h, again irrespective of the order of administration of the two drugs.

### *BLM* in vivo/*PENT* in vitro

The results of 2 experiments in which cell suspensions were prepared from tumours and exposed *in vitro* to PENT for

TABLE I.—*Effect of Delayed Subculture on Surviving Fraction of Cells Exposed* In Vitro *to BLM Alone or BLM and PENT in Combination*

| Cell growth phase | Experiment no. | BLM alone | | BLM + PENT | |
|---|---|---|---|---|---|
| | | SF | SFR(4) | SF | SFR(4) |
| Exponential | 1 | 0·17 | 3·8 | 0·0025 | 1·2 |
| | 2 | 0·59 | 0·8 | 0·0032 | 2·6 |
| | 3 | 0·14 | 2·2 | 0·00053 | 5·1 |
| Early plateau | 1 | 0·42 | 1·2 | 0·0072 | 7·6 |
| | 2 | 0·37 | 1·8 | 0·0131 | 6·3 |
| | 3 | 0·16 | 2·8 | 0·0043 | 3·5 |
| Late plateau | 1 | 0·043 | 7·0 | 0·30 | 1·1 |
| | 2 | 0·230 | 1·9 | 0·40 | 2·0 |
| | 3 | 0·066 | 6·7 | 0·044 | 9·1 |

SF = Surviving fraction for immediate subculture

$$\text{SFR(4)} = \frac{\text{Surviving fraction for 4 h subculture delay}}{\text{Surviving fraction for immediate subculture}}$$

FIG. 3.—Change in surviving fraction of EMT6 cells in exponential phase exposed to BLM (B) and PENT (P) each for 1 h with various intervals between exposures. " Together " means simultaneous exposure. Zero interval indicates one drug followed immediately by the other. Circles and triangles show 2 separate experiments. Error bars indicate ±2 standard errors based on the total colony count on groups of 4 replicate dishes.

30 min are shown in Table II. The tumours themselves were excised from mice at various times after BLM administration. It may be seen that significant reduction in surviving fraction was produced especially for tumours removed at 30 min and 2 h after BLM administration. The reduction for tumours taken at 6 h after BLM was however much less.

### *BLM + PENT* in vivo

Results of experiments in which the two drugs were each administered to tumour-bearing mice are shown in Table III. The dose of PENT used was 20 mg/kg. The range of values obtained for the ratio of effect with PENT to effect without PENT was wide, but within the normal range seen for this type of assay. The initial effect of BLM (at 30 min) was of the same order of magnitude in the presence or absence of PENT, and the large recovery seen with delayed subculture from 30 min to 6 h still occurred. It did not appear that PENT alone is very cytotoxic to the tumour cells *in vivo* even at this very high dose level, *i.e.* in excess of the $LD_{50}$.

### DISCUSSION

It does not appear likely from these results that the combination of BLM and PENT will be clinically useful in tumour therapy. Firstly, PENT itself is highly toxic and also insoluble in water or alcohol with subsequent problems of administration. Secondly, both the initial response of the tumour cells to

TABLE II.—*Effect of 30 min Incubation with Pentamycin on Surviving Fraction of Cells Taken from Solid EMT6 Tumours at Various Times after BLM Administration* in Vivo

| Experiment | Surviving fractions: Control | | 30 min after BLM | | 2 h after BLM | | 6 h after BLM | |
|---|---|---|---|---|---|---|---|---|
| | −PENT | +PENT | −PENT | +PENT | −PENT | +PENT | −PENT | +PENT |
| A | 1·0 | 0·99 | $3·45\times10^{-3}$ | $1·07\times10^{-3}$ | $6·34\times10^{-2}$ | $1·01\times10^{-3}$ | 0·72 | 0·44 |
| | +/− =0·99 | | +/− =0·31 | | +/− =0·16 | | +/− =0·61 | |
| B | 1·0 | 0·84 | $5·6\times10^{-3}$ | $1·3\times10^{-3}$ | $2·0\times10^{-2}$ | $8·2\times10^{-3}$ | 0·78 | 0·62 |
| | +/− =0·84 | | +/− =0·23 | | +/− =0·41 | | +/− =0·80 | |

TABLE III.—*Effect of BLM + PENT* in Vivo *upon EMT6 Solid Tumours*

| Experiment | Surviving fractions: 30 min after BLM | | 2 h after BLM | | 6 h after BLM | | PENT alone 2 h | PENT alone 6 h |
|---|---|---|---|---|---|---|---|---|
| | −PENT | +PENT | −PENT | +PENT | −PENT | +PENT | | |
| A | 0·0020 | 0·0012 | 0·018 | 0·024 | 0·30 | 0·14 | 0·75 | 0·58 |
| | +/− = 0·60 | | +/− = 1·33 | | +/− = 0·47 | | | |
| B | 0·0013 | 0·0015 | — | | 0·20 | 0·37 | 0·84 | 0·83 |
| | +/− = 1·14 | | | | +/− = 1·85 | | | |
| C | 0·00096 | 0·0030 | 0·029 | 0·017 | 0·26 | 0·26 | — | — |
| | +/− = 3·1 | | +/− = 0·58 | | +/− = 1·0 | | | |

BLM and their subsequent recovery do not appear to be greatly influenced by PENT. One possible reason for this is that the drug does not get to the tumour cells following this mode of administration. If this is the case, however, it seems likely that the non-access is absolute rather than dependent upon a particular timing of the combination. Our *in vitro* results indicate that exposure of cells to PENT within the period of several hours each side of the BLM exposure can cause potentiation, and hence the actual timing should not be too important *in vivo.*

The response of tumour cells treated *in vitro* following preparation of suspension is instructive in this respect in that only limited potentiation is produced in a situation where access of PENT to the cells is without doubt. It appears therefore that at least a large proportion of the total population of tumour cells react to the drug combination in the same way as late plateau phase cells *in vitro.*

The idea that PENT acts by increasing the amount of BLM which penetrates into the cell (Nakashima *et al.*, 1974) may well explain our findings. It has been reported by Fujimoto (1974) that ascites tumour cells exposed to $^{14}C$ BLM showed the label absorbed to the cell membrane at 2 h after drug administration with subsequent movement of the label to the nuclear membrane by 4 h. It seems possible, therefore, that cells exposed to PENT undergo some change in the membrane which allows more penetration of BLM. If cells are exposed to PENT after BLM then BLM which is attached in some way to the cell membrane and which normally would be harmlessly released at some later time is allowed to penetrate the membrane.

If the concept that PENT acts by altering membrane permeability is correct, then our results imply that the relative response to BLM alone shown by cells in various growth phases is highly dependent upon the state of the membrane during these phases, and that the cell membrane is a highly resistant barrier to BLM at all growth phases *in vitro.*

Carried further, it is also possible that the " repair of potentially lethal damage " phenomenon could be accounted for in terms of membrane permeability. It would be necessary to postulate that during the subculture procedure, or else during some process occurring shortly afterwards, BLM is allowed to pass from its membrane-absorbed site into the cell where cytotoxicity occurs. Cells left without subculture would release the BLM from the membrane into the extracellular fluid without cytotoxicity occurring. We are intending to investigate these possibilities in our laboratory using labelled BLM.

### REFERENCES

FUJIMOTO, J. (1974) Radioautographic Studies on the Intracellular Distribution of Bleomycin-$^{14}C$ in Mouse Tumor Cells. *Cancer Res.*, **34**, 2969.

KUWANO, M., KAMIYA, T., ENDO, H. & KOMIYAMA, S. (1973) Potentiation of 5-Fluorouracil, Chromomycin A3, and Bleomycin by Amphotericin B of Polymyxin B in Transformed Fibroblastic Cells. *Antimicrob. Agents Chemother.*, **3**, 580.

MEDOFF, G., VALERIOTE, F., LYNCH, R. G., SCHLESSINGER, D. & KOBAYASHI, G. S. (1974) Synergistic Effect of Amphotericin B and 1,3-Bis(2-chloroethyl)-1-nitrosourea against a Transplantable AKR Leukemia. *Cancer Res.*, **34**, 974.

NAKASHIMA, T., KUWANO, M., MATSUI, K., KOMIYAMA, S., HIROTO, I. & ENDO, H. (1974) Potentiation of Bleomycin by an Antifungal Polyene, Pentamycin, in Transformed Animal Cells. *Cancer Res.*, **34**, 3258.

TERASIMA, T., TAKABE, Y., KATSUMATA, T., WATANABE, M. & UMEZAWA, H. (1972) Effect of Bleomycin on Mammalian Cell Survival. *J. natn. Cancer Inst.*, **49**, 1093.

TWENTYMAN, P. R. & BLEEHEN, N. M. (1974) The Sensitivity to Bleomycin of a Solid Mouse Tumour at Different Stages of Growth. *Br. J. Cancer*, **30**, 469.

TWENTYMAN, P. R. & BLEEHEN, N. M. (1975*a*) Changes in Sensitivity to Radiation and to Bleomycin Occurring during the Life-history of Monolayer Cultures of a Mouse Tumour Cell Line. *Br. J. Cancer*, **31**, 68.

TWENTYMAN, P. R. & BLEEHEN, N. M. (1975*b*) Studies of "Potentially Lethal Damage" in EMT6 Mouse Tumour Cells Treated with Bleomycin either *in Vitro* or *in Vivo*. *Br. J. Cancer*, **32**, 491.

TWENTYMAN, P. R., WATSON, J. V., BLEEHEN, N. M. & ROWLES, P. M. (1975) Changes in Cell Proliferation Kinetics Occurring During the Life-history on Monolayer Cultures of a Mouse Tumour Cell Line. *Cell Tissue Kinet.*, **8**, 41.